# Exploratory Incisions

**Published:** 1899-03-04

**Authors:** 


### EXPLORATORY INCISIONS.

In reference to the above case it is not uninteresting to note that at a recent meeting of the New York Academy of Medicine, Dr. Carl Beck[1] showed a femoral aneurysm which he had dissected out, and the nature of which had also not been diagnosed until it was cut into. The patient had had an injury three years before, but it caused him no trouble until three months before he was seen, when he noticed a tumour the size of a lemon, about the middle of the thigh, at the point of injury. The tumour increased rapidly in size, and as there was slight fluctuation it was thought to be an abscess, and an incision was seriously considered. Fortunately this was not attempted. When the patient was seen by Dr. Beck there was a large ovoid tumour extending from the internal condyle of the femur to the groin. It was very hard, and no fluctuation could be felt. The leg and foot were absolutely normal. At first it was thought to be an osteo-sarcoma, its hardness, slow growth, and immobility being in favour of that assumption. Two exploratory incisions were made, and the tumour was found to contain blood, examination of which showed an abundance of leucocytes. But a skiagram showed the femur to be normal, so that diagnosis was eliminated. The tumour was then thought to be a sarcoma of the sheath of the femoral vein, and operation was undertaken. A gush of blood, however, followed division of the tissues, and further examination showed the abnormality to be an aneurysm. Forcible pressure was made to control hæmorrhage until

[1] Med. News, Feb. 18.

the femoral artery was tied in Scarpa's triangle, and then the sac was dissected out with ease. The two cases are very interesting, as illustrating the difficulties which sometimes attend the diagnosis of tumours even when all modern aids are at our disposal. They also suggest that perhaps we might get a little further than we have done in the details of that useful but somewhat crude proceeding, an exploratory incision. No doubt "seeing is believing," but we fancy that any surgeon who has experienced the misfortune will agree that to cut into an aneurysm is a singularly unfortunate method of diagnosis. Yet here, almost at the same time, we come haphazard upon two cases in which this very thing has been done, and done by men of the greatest possible experience. It may be worth while, then, to suggest that cutting into a lump is a proceeding which does not always give very accurate information as to its nature. Dissecting round a tumour may, however, sometimes be useful, and it is possible that much may in some cases be learned by an exploratory incision even without cutting into a tumour at all. In an exploratory laparotomy the very first thing done is to ascertain the relations of the tumour and its connection with other parts, and it might be worth while in other departments of surgery to bear in mind that an exploratory incision is made with the object of exploring the tumour and its relations, but not necessarily of exploring its contents.

